# Supplementary figures and images for: Novel Cell Models to Study Myelin and Microglia Interactions
Source: Int J Mol Sci. 2025 Feb 28;26(5):2179. doi: 10.3390/ijms26052179 (PMC11900003; doi:10.3390/ijms26052179)

Figure s1

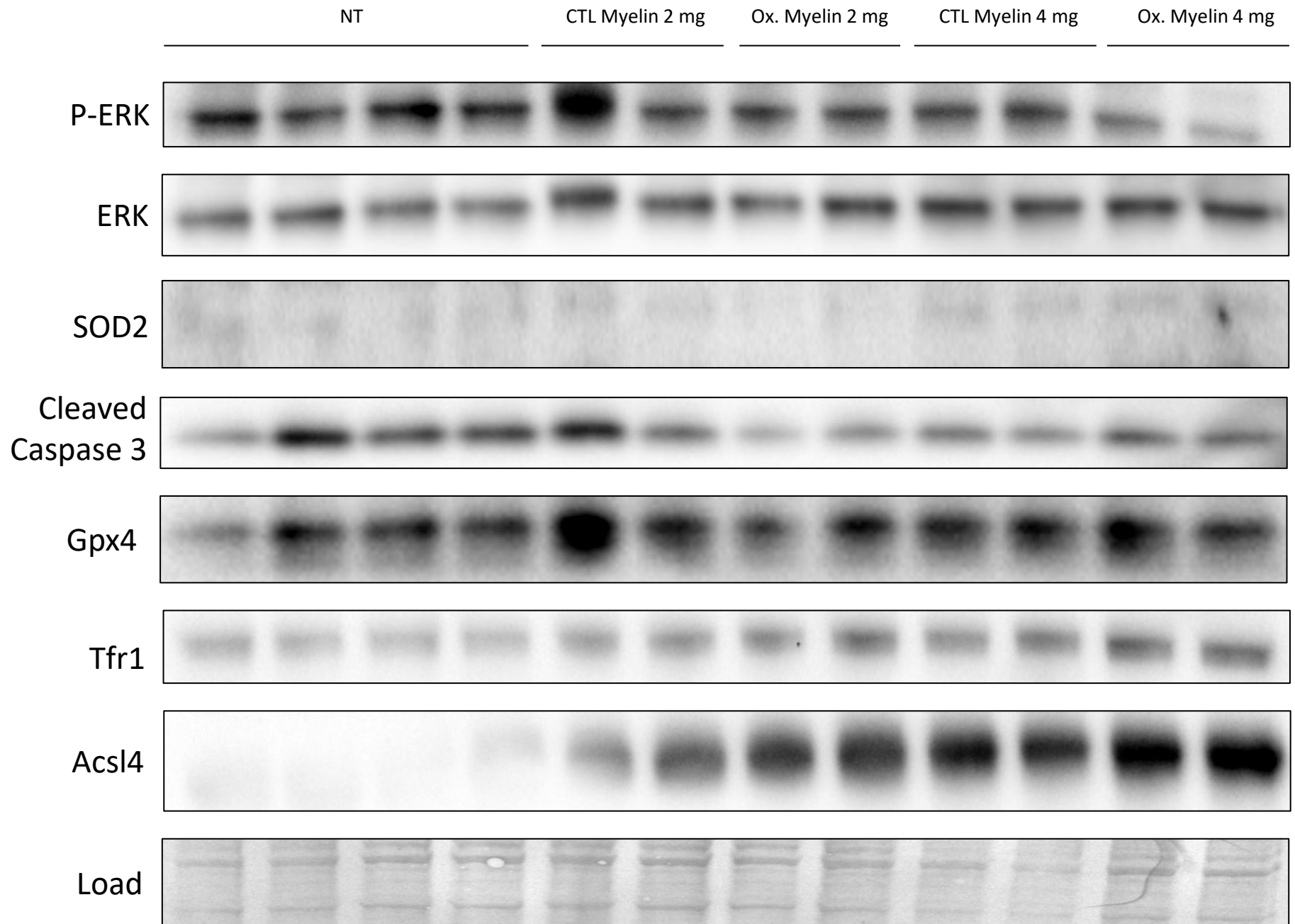

Figure s2

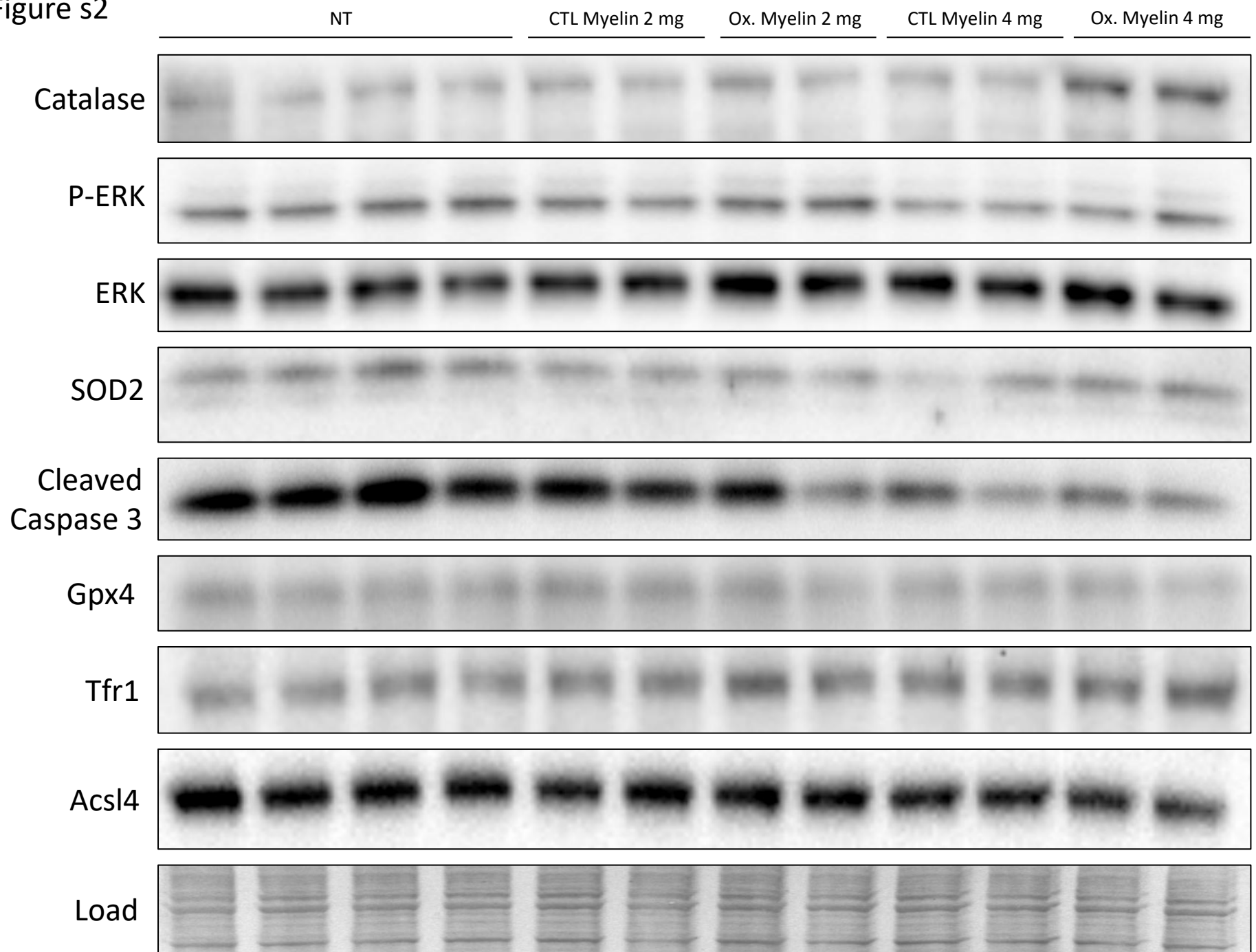

# Figure s3

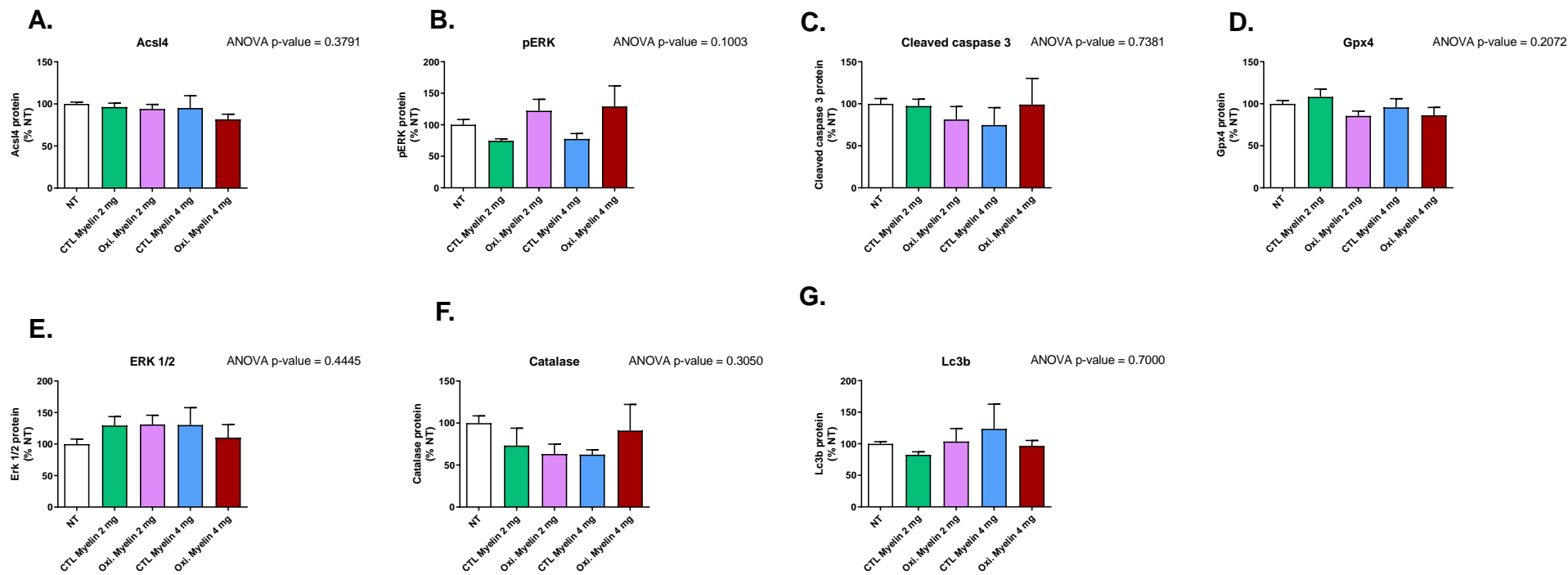

Supplement: Supplementary file 1 [file ijms-26-02179-s001.zip › ijms-3476482-supplementary.pdf]
